# Negative Affect and Problematic Binge-Watching: The Mediating Role of Unconstructive Ruminative Thinking Style

**DOI:** 10.5334/pb.1163

**Published:** 2022-09-30

**Authors:** Pauline Billaux, Joël Billieux, Leonie Gärtner, Pierre Maurage, Maèva Flayelle

**Affiliations:** 1 Institute of Psychology, University of Lausanne, Lausanne, Switzerland; 2 Louvain Experimental Psychopathology research group (LEP), Psychological Science Research Institute, UCLouvain, Louvainla-Neuve, Belgium; 3 Institute for Health and Behaviour, Department of Behavioural and Cognitive Sciences, University of Luxembourg, Esch-sur-Alzette, Luxembourg

**Keywords:** Binge-watching, rumination, process-based approach, emotion regulation strategy, behavioural addiction

## Abstract

The practice of binge-watching (i.e., watching multiple episodes of TV series in one session) has become increasingly prevalent, but comprehending its nature and potential underlying factors has been challenging. In particular, problematic binge-watching remains ill-defined and conceptualized, being regarded either as an addictive behaviour or a maladaptive emotion regulation strategy. Following a process-based approach, in the current study we explored the latter conceptualization, by investigating the potential mediating role of an unconstructive ruminative thinking style between negative affect and problematic binge-watching. To this end, TV series viewers completed an online survey assessing socio-demographic variables, TV series viewing habits, binge-watching motives and engagement, ruminative thinking styles and affect. Based on their answers, participants were allocated to one of the following three groups: non-binge-watchers (n = 59), trouble-free binge-watchers (n = 85), or problematic binge-watchers (n = 162). Group comparisons and mediation analyses were conducted to explore the underlying role of unconstructive rumination in problematic binge-watching. Results showed that, apart from the pattern of TV series watching, trouble-free binge-watchers shared little to no similarity with problematic binge-watchers, therefore supporting the need to differentiate these two behaviours. Moreover, mediation analyses revealed that an unconstructive ruminative thinking style partially mediated the relationship between negative affect and coping/escapism and that it fully accounted for the relationship between negative affect and binge-watching derived positive emotions in problematic binge-watchers. These findings thus add to the notion that problematic binge-watching might serve as a way to bolster a maladaptive emotion regulation strategy, implying that unconstructive rumination acts as a mediating process in this context.

## Introduction

The continuous development and improvement of on-demand streaming platforms providing unlimited access to a wide array of content (e.g., Netflix, Hulu or Amazon Prime) has promoted a new pattern of TV series consumption called binge-watching (i.e., watching multiple TV series episodes in one session; Flayelle et al., 2020a; Starosta & Izydorczyk, 2020). Between 2015 and 2020, binge-watching (together with serial viewing; i.e., watching series over multiple days, weeks or months) strongly increased at the expense of traditional appointment viewing (i.e., watching an episode each week, when aired) (Rubenking & Bracken, 2021) and ultimately became the new normative way to watch TV series (Business Wire, 2019; Statista, 2020).

In parallel with this expansion, a growing body of research has explored the phenomenology and correlates (e.g., psychological, personality or psychiatric factors) of binge-watching, generating an emerging area of scientific inquiry and promoting debates regarding how to define, assess and conceptualize this new media-related behaviour (Flayelle et al., 2020a; Starosta & Izydorczyk, 2020). Some scholars have notably conceptualized excessive binge-watching as a potential addictive behaviour (e.g., Forte et al., 2021; Orosz et al., 2016; Tóth-Király et al., 2017), as it not only shares phenomenological characteristics with substance use disorders at the symptomatic level (e.g., tolerance; Orosz et al., 2016), but it is also associated with a set of physical (e.g., obesity due to its associated sedentary lifestyle; Spruance et al., 2017) and psychological (e.g., emotional distress; Granow et al., 2018; Shim et al., 2018) outcomes. Such conceptualization of binge-watching is rooted in a confirmatory framework that posits, *a priori*, an addictive overtone to excessive behaviours, provided that symptomatic similarities with substance use disorders are detected (Billieux et al., 2015a; Flayelle et al., 2022). In this framework, those behaviours are considered ‘behavioural addictions’ and analysed through the lens of substance use disorder models (Starcevic et al., 2018). However, this conceptualization involves the risk of overpathologizing everyday behaviours (Billieux et al., 2015a), as it often results in overlooking the crucial distinction between intensive—but healthy—involvement in appetitive and rewarding activities and intensive—yet problematic—involvement (i.e., associated with negative consequences and functional impairment; Billieux et al., 2019; Kardefelt-Winther et al., 2017). Such overpathologization is especially due to a widespread approach that consists of recycling substance use disorder criteria (e.g., tolerance or preoccupation) to assess problematic involvement levels in specific activities (e.g., TV series watching), despite the fact that such recycled criteria might have low clinical validity and utility, or poor prognostic value when applied to behavioural addictions (Billieux et al., 2019; Castro-Calvo et al., 2021; Flayelle et al., 2022). A sound way to address this issue is to abandon the mere symptom-based focus and apply a process-based approach to the study of behavioural addictions (Billieux et al., 2015a; 2015b; Bonnaire & Billieux, 2022; Flayelle et al., 2022). Process-based approaches posit that key psychological processes mediate the relationship between multi-determined risk factors (i.e., biological, social and circumstantial) and psychopathological symptoms or syndromes, therefore implying that psychological interventions should target those processes (Kinderman, 2005; Kinderman & Tai, 2007). Applying such a process-based approach to binge-watching has the potential to unveil its underlying psychological mechanisms (Flayelle et al., 2017; 2019a). Similar to other types of potentially excessive behaviours, binge-watching shows a dual nature in terms of its associated outcomes (Flayelle et al., 2019b; 2020a). Aside from its documented negative consequences, binge-watching is also associated with positive outcomes such as enhanced socialization (Flayelle et al., 2017; Rubenking & Bracken, 2021), enjoyment (Granow et al., 2018; Merrill & Rubenking, 2019) and engagement (e.g., intensity of parasocial relationships with fictional characters and narrative transportation; Erickson et al., 2019). Binge-watching should thus be conceptualized as a two-faceted phenomenon that reflects either problematic or non-harmful involvement in TV series watching (Flayelle et al., 2019b; Ort et al., 2021). Given the duality of binge-watching, evidence of daily life interference and functional impairments should be present in order to qualify binge-watching as genuinely problematic or dysfunctional, similar to what has already been suggested in relation to other types of potentially excessive behaviours (e.g., Kardefelt-Winther et al., 2017).

In support for the aforementioned dichotomy, motives and engagement were shown to dissociate problematic from healthy involvement in binge-watching (Flayelle et al., 2019b; 2019c; 2020a; Ort et al., 2021). Although problematic binge-watchers mostly report negative reinforcement motives (e.g., escapism), healthy binge-watchers tend to report more hedonistic motives (e.g., entertainment) (Flayelle et al., 2019b; Sung et al., 2018). Moreover, research shows that problematic binge-watchers report more negative affect than healthy binge-watchers (Flayelle et al., 2019b) and that problematic binge-watching is associated with symptoms of depression and anxiety (Starosta et al., 2021). Such evidence led to the proposal that the drive for problematic binge-watching might mainly lie in the urge to reduce unpleasant emotional states, this behaviour potentially constituting a maladaptive coping or emotion regulation strategy (e.g., Flayelle et al., 2019a; 2020a; Rubenking & Bracken, 2018; Sigre-Leirós et al., 2022).

Rumination is a classic marker of emotion dysregulation and a transdiagnostic process involved in the onset and maintenance of a wide array of mental disorders (e.g., Nolen-Hoeksema et al., 2008). In such perspective, and following the reasoning proposed by Kinderman (2005) in his process-based model of mental disorders, rumination may thus constitute a key psychological process explaining the association between negative affect (i.e., risk factor) and problematic binge-watching (i.e., symptom), in that the positive influence of negative affect on the emergence and development of problematic binge-watching might be bolstered by a higher level of rumination. Rumination is a multifaceted construct (Smith & Alloy, 2009), conceptualized as repetitive and prolonged thinking about the causes and consequences for one’s situation, feelings and experiences (Nolen-Hoeksema et al., 2008; Watkins, 2008). The mediating role of rumination in various problematic online behaviours has already been established through a series of studies that focused on the problematic use of social media (Dempsey et al., 2019; Mitra & Rangaswamy, 2019), video games (Kökönyei et al., 2019) and smartphones (Billieux et al., 2015b; Elhai et al., 2018; 2020a; Liu et al., 2017). Previous research conducted from a process-based perspective has also demonstrated that rumination can be viewed as a psychological process mediating the relationship between specific risk factors (i.e., familial history of mental health disorders, negative life events, social deprivation) and the onset of anxiety and depression (Kinderman et al., 2013; 2015). In line with these results, and because 1) problematic binge-watchers report more negative affect than do healthy binge-watchers (Flayelle et al., 2019b) and 2) problematic binge-watching might constitute a maladaptive coping strategy to regulate negative emotional states (e.g., Flayelle et al., 2019a; 2020a; Rubenking & Bracken, 2018), we hypothesized a mediating role of rumination (i.e., hypothesized disturbed psychological process) between negative affect (i.e., a hypothesized risk factor) and the development of problematic binge-watching (i.e., a hypothesized resulting symptom), thereby following a process-based approach (Kinderman, 2005; Kinderman & Tai, 2007).

Yet, and critically, rumination is established as a multifaceted construct (Smith & Alloy, 2009) comprising both constructive and non-constructive aspects (Watkins, 2008). A recent framework proposed by Philippot et al. (2021) differentiates between three dimensions: 1) analytic evaluative repetitive thinking (AERT), 2) concrete experiential repetitive thinking (CERT) and 3) creative dendritic repetitive thinking (CDRT). AERT refers to unconstructive abstract thoughts related to one’s mood or situation. They are oriented towards the past and future and are associated with anxious and depressive moods (Douilliez et al., 2014; Philippot et al., 2021). Conversely, CERT and CDRT both refer to constructive thoughts related to efficient emotion regulation (Philippot et al., 2021). Traditionally, rumination operationalization follows dual-based reasoning in which AERT is an unconstructive type of rumination, since it is, overall, associated with negative health outcomes (e.g., depression), whereas CERT constitutes a constructive type of rumination, since it is associated with positive health outcomes (e.g., traumatic experience recovery) (Watkins, 2008). Recently, Philippot et al. (2021) suggested that CERT does not constitute a single latent construct but rather reflects two distinct factors, leading the authors to suggest a new three-factor model of rumination (i.e., AERT for unconstructive rumination, CERT and CDRT for constructive rumination). CERT is related to concrete present affect and situations, whereas CDRT is not anchored to a specific time frame, being characterized by flexible, creative content. Therefore, whereas CERT content is centred on concrete problem-solving, CDRT is instead related to the generation of original ideas (Philippot et al., 2021).

### Current study

In this study, we aimed to test the mediating role of ruminative thinking styles between negative affect and problematic binge-watching. In line with previous work (Flayelle et al., 2020b), we established three groups of TV series viewers: a) *non-binge-watchers* (NBWs), b) *trouble-free binge-watchers* (TBWs) and c) *problematic binge-watchers* (PBWs). From previous binge-watching research (Flayelle et al., 2019b; 2020a), we expected that (1a) PBWs would differ from TBWs on binge-watching-related motives (i.e., PBWs reporting more negative reinforcement motives such as coping/escapism) and engagement (i.e., PBWs reporting features such as loss of control or negative consequences) and that (1b) PBWs would report more negative affect than both NBWs and TBWs. From the triadic conceptualization of rumination (Philippot et al., 2021), we then expected PBWs to principally report AERT (i.e., unconstructive rumination associated with negative health outcomes), and NBWs and TBWs to mainly report CERT and CDRT (i.e., constructive rumination associated with positive health outcomes). Assuming a process-based model of psychopathology (Kinderman, 2005; Kinderman & Tai, 2007), we postulated that, in PBWs, AERT would constitute a mediator psychological process between negative affect and symptom severity.

## Methods

### Participants and procedure

We used Qualtrics survey software to conduct this online study, and disseminated it to members of French-speaking TV series fan communities through social networks (i.e., Facebook) and to French-speaking university students from Belgium, France and Luxembourg. Participants were informed of the study objectives and provided their consent prior to survey completion (lasting approximately 20 minutes). The survey comprised, in fixed order, questions assessing socio-demographic variables and TV series viewing habits, and then the French versions of the Watching TV Series Motives Questionnaire (WTSMQ; Flayelle et al., 2019c, 2020c) and Binge-Watching Engagement and Symptoms Questionnaire (BWESQ; Flayelle et al., 2019c, 2020c), the Repetitive Thinking Mode Questionnaire (RTMQ; Philippot et al., 2021) and the Positive and Negative Affect Schedule (PANAS; Gaudreau et al., 2006).

Inclusion criteria for this study were 1) being at least 18 years old, 2) being fluent in French and 3) having watched TV series episodes regularly or intensively (i.e., several episodes in one session) through DVD, USB, or subscription video on demand (SVOD) devices or through streaming platforms over the last six months. We conducted this study in accordance with the Declaration of Helsinki. We ensured anonymity and confidentiality throughout survey completion. A prize drawing allowed 10 participants to win a gift voucher of 15 Euros. All data and study materials are available via the Open Science Framework at https://osf.io/s9y26/.

### Measures

#### Socio-demographic information and TV series viewing habits

We recorded socio-demographic variables (i.e., age, educational level, French level, gender and marital status) and TV series viewing habits (i.e., devices used, number of episodes watched in one session, time spent per viewing session during weekdays and days off, watching frequency, reported feeling of TV series watching dependency, presence of functional impact and problematic binge-watching^1^). TV series viewing habits data were used to form the three groups (i.e., NBWs, TBWs and PBWs) in accordance with the selection criteria of Flayelle et al. (2020b) as reported in Table 1. Inclusion criteria for the binge-watching groups were 1) spending at least two hours watching TV series per session and 2) watching at least three episodes consecutively. The rationale for using such quantitative thresholds is that binge-watching is usually defined in the literature as watching at least three “hour-long” (average length: 42 minutes) TV series episodes in one sitting (e.g., Erickson et al., 2019; Merril & Rubenking, 2019). Exclusion criteria for NBWs and TBWs were 1) reporting a functional impact and 2) identifying as a problematic binge-watcher. PBWs had to report a functional impact due to binge-watching but self-identification as a problematic binge-watcher was neither an inclusion nor an exclusion criterion for this group.

**Table 1:** Selection criteria for the three groups of participants.


	NON-BINGE-WATCHERS (NBWs)	TROUBLE-FREE BINGE-WATCHERS (TBWs)	PROBLEMATIC BINGE-WATCHERS (PBWs)

***Time spent watching per viewing session***	<120 minutes	≥120 minutes	≥120 minutes

***Number of episodes watched in a row***	<3	≥3	≥3

***Reported functional impact***	No	No	Yes

***Self-identification as problematic TV series viewer***	No	No	Yes or No


*Note*. We derived these criteria from Flayelle et al. (2020b). Accordingly, participants reported their average time spent watching TV series (in minutes, during the weekends and weekdays), the number of episodes typically watched in one sitting, and whether 1) binge-watching negatively affected their everyday life and 2) they considered their TV series consumption as problematic.

#### Watching TV Series Motives Questionnaire (WTSMQ)

The WTSMQ (Flayelle et al., 2019c; 2020c) measures motives for TV series watching. It comprises 22 items scored on a 4-point Likert scale from 1 (*not at all*) to 4 (*to a great extent*). The WTSMQ assesses four main motivational dimensions: *coping/escapism* (eight items, e.g., ‘*I watch TV series to overcome loneliness*’), *emotional enhancement* (five items, e.g., ‘*I watch TV series to feel strong emotions like the excitement or the thrill they give me*’), *enrichment* (five items, e.g., ‘*I watch TV series because they give me food for thought on a number of subjects*’) and *social* (four items, e.g., ‘*I watch TV series to relate to others more easily, because TV series give me something to discuss*’). We computed average scores for each motive. The Cronbach’s alpha values obtained in the current sample ranged from .64 (social) to .82 (coping/escapism).

#### Binge-Watching Engagement and Symptoms Questionnaire (BWESQ)

The BWESQ (Flayelle et al., 2019c; 2020c) assesses binge-watching engagement and symptoms of problematic binge-watching. It comprises 40 items, scored on a 4-point Likert scale from 1 (*not at all*) *to 4* (*to a great extent*). It evaluates seven factors: *binge-watching* (six items, e.g., ‘*I always need to watch more episodes to feel satisfied*’), *dependency* (five items, e.g., ‘*I am usually in a bad mood, sad, depressed or annoyed when I can’t watch any TV series, and I feel better when I am able to watch them again*’), *desire/savouring* (six items, e.g., ‘*I get really excited when a new episode is released*’), *engagement* (eight items, e.g., ‘*In my opinion, TV series are a part of my life and they contribute to my welfare*’), *loss of control* (seven items, e.g., ‘*I watch more TV series than I should*’), *pleasure preservation* (three items, e.g., ‘*I worry about getting spoiled*’) and *positive emotions* (five items, e.g., ‘*Watching TV series is a cause for joy and enthusiasm in my life*’). We computed an average score for each factor. The Cronbach’s alpha values obtained in the current sample ranged from .61 (pleasure preservation) to .82 (loss of control).

#### Repetitive Thinking Mode Questionnaire (RTMQ)

The RTMQ (Philippot et al., 2021) is an 18-item scale assessing ruminative thinking style, according to the triadic conceptualization of rumination (i.e., AERT, CERT and CDRT). Each item is scored on a 4-point Likert scale from 1 (*almost never*) to 4 (*almost always*). Each type of rumination is assessed through six items. AERT is an unconstructive type of rumination. AERT contents are overgeneralized and abstract, and they relate to the causes and consequences of one’s situation (e.g., ‘*I feel under pressure to prevent my worst fears from happening*’). In contrast, CERT and CDRT both represent a constructive type of rumination. However, whereas CERT contents relate to concrete, present experiences (e.g., ‘*I am very focused on what is happening inside me*’), CDRT contents are flexible and creative, similar to mind-wandering (e.g., ‘*My mind is constantly shifting from an idea to a new idea*’). The Cronbach’s alpha values in the current sample ranged from .68 (CERT) to .79 (CDRT).

#### Positive and Negative Affect Schedule (PANAS)

The PANAS (French validation: Gaudreau et al., 2006) is a 20-item mood scale assessing both positive (10 items, e.g., ‘*enthusiastic*’) and negative (10 items, e.g., ‘*scared*’) affect (Watson et al., 1988). Items are scored on a 5-point Likert scale from 1 (*very slightly or not at all*) to 5 (*extremely*). The PANAS can be used to assess state (e.g., ‘*you feel this way right now, that is, at the present moment*’), mood (e.g., ‘*you have felt this way during the past few weeks*’) or trait (e.g., ‘*you generally feel this way, that is, how you feel on average*’) measures of affect (Gaudreau et al., 2006; Watson et al., 1988). Because we explored the overall relationship between negative affect and binge-watching patterns, we relied on the trait version. For each subscale, we computed a total score by summing item scores. The Cronbach’s alpha values in the current sample ranged from .72 (positive affect) to .85 (negative affect).

### Data reduction and statistical analyses

We followed a listwise deletion approach and excluded data from participants who did not respond to all questions, reducing the sample from 509 to 420 participants. We used TV series watching habits data (i.e., time spent watching TV series and number of episodes watched in a row during typical viewing sessions, reported functional impact and self-identification as a problematic binge-watcher) to form the NBWs, TBWs, and PBWs groups. This led to the additional exclusion of 114 participants who did not meet the specific criteria used to create the three groups of interest for our study (see Table 1). The final sample (see Table 2) thus comprised 306 participants (female = 81.70%; M_age_ = 25.40; SD_age_ = 7.88) of NBWs (n = 59), TBWs (n = 85) and PBWs (n = 162).

**Table 2:** Socio-demographic and TV series viewing characteristics of the three groups.


	NBWs (N = 59)	TBWs (N = 85)	PBWs (N = 162)

**Socio-demographics**			

Age, M (SD)	26.63 (8.68)	26.27 (8.66)	24.49 (7.05)

Gender – female, N (%)	44 (74.60)	72 (84.70)	134 (82.70)

**Binge-watching habits**			

Reported functional impact, N (%)	/	/	162 (100)

Self-identification as a problematic viewer, N (%)	/	/	40 (24.70)

Time spent watching (minutes) per weekday, M (SD)	50.93 (26.77)	140.85 (86.35)	129.35 (88.40)

Time spent watching (minutes) per day off, M (SD)	98.12 (50.88)	259.93 (117.30)	260.48 (129.01)

1 episode per session, N (%)	10 (16.90)	/	/

2 episodes per session, N (%)	49 (83.10)	/	/

3 episodes per session, N (%)	/	33 (38.80)	70 (43.20)

4 episodes per session, N (%)	/	28 (32.90)	33 (20.40)

5 episodes per session, N (%)	/	8 (9.40)	20 (12.30)

6 episodes per session, N (%)	/	14 (16.50)	32 (19.80)

>6 episodes per session, N (%)	/	2 (2.40)	7 (4.30)


*Note*. NBWs: non-binge-watchers; TBWs: trouble-free binge-watchers; PBWs: problematic binge-watchers.

All statistical analyses were performed with SPSS 27.0 (IBM, Corp.) using a significance level of alpha .05 (bilateral). First, preliminary statistical analyses on socio-demographic and binge-watching habits variables were conducted. We then performed chi-squared tests, Kruskal-Wallis tests and one-way analysis of variance (ANOVA) to explore group differences on these variables. The matrix of correlation (Spearman) between all variables is available in the Open Science Framework: https://osf.io/s9y26/. We next conducted one-way and Welch’s ANOVAs with each of the WTSMQ, BWESQ, RTMQ and PANAS subscales as the within-subject factor and group (NBWs, TBWs, PBWs) as the between-subject factor. When significant main effects emerged, ANOVAs were followed by Tukey’s or Games-Howell post hoc tests. Finally, to test our main hypothesis derived from the process-based framework (Kinderman, 2005; Kinderman & Tai, 2007), we conducted mediation analyses on PBWs (n = 162), with dependent variables deriving from previous ANOVA results (i.e., each facet of the BWESQ and WTSMQ for which PBWs significantly differed from the other two groups). As recommended for a small sample (Preacher & Hayes, 2004), we carried out mediation analyses through the bootstrapping method (2000 bootstrapped samples) to test indirect effects (axb).

## Results

### Socio-demographic information and TV series viewing habits

The groups did not differ significantly for age (F_Welch_ (2, 129.83) = 2.25, *p* = .11, η^2^ = .02), educational level (χ^2^(2) = 4.29, *p* = .12), French level (χ^2^(2) = 1.18, *p* = .56), gender (χ^2^(6) = 7.15, *p* = .31) and marital status (χ^2^(10) = 14.16, *p* = .17) (for a comprehensive summary of descriptive statistics, see supplementary results available at https://osf.io/s9y26/). NBWs differed significantly from TBWs and PBWs on binge-watching habits variables, scoring lower on the frequency of watching two episodes in one sitting (χ^2^(2) = 27.44, *p* < .001), hours spent watching during both weekdays (F_Welch_(2, 175.73) = 78.83, *p* < .001, η^2^ = .15) and days off (F_Welch_(2, 180.16) = 122.73, *p* < .001, η^2^ = .24) and number of episodes watched in one session (χ^2^(2) = 150.65, *p* < .001). In addition, the three groups differed significantly for reported feeling of dependency on TV series watching [χ^2^(2) = 24.00, *p* < .001], with the highest reported proportion in PBWs (38.90) and the lowest in NBWs (6.80).

### Outcome measures

#### TV series watching motives

Regarding motive variables, a main effect of group was found for coping/escapism (F_Welch_(2, 156.14) = 22.08, *p* < .001, η^2^ = .12) and emotional enhancement (F(2, 303) = 3.86, *p* = .02, η^2^ = .03). PBWs scored on average significantly higher than both NBWs and TBWs on the coping/escapism facet, whereas the only significant average score difference between groups on the emotional enhancement facet was observed between PBWs and NBWs (with a higher mean score for PBWs). No main effect of group was found for enrichment (F(2, 303) = .60, *p* = .55, η^2^ = .00) or social (F(2, 303) = 2.56, *p* = .08, η^2^ = .02) motivational facets. These results are reported in Table 3.

**Table 3:** Post-hoc tests conducted following significant ANOVAs.


COMPARISON		MEAN DIFFERENCE (I–J)	STANDARD ERROR	*p*-VALUE	95% CONFIDENCE INTERVAL

GROUP (I)	GROUP (J)				

**Tukey’s test**.					

BWESQ – binge-watching					

NBWs	TBWs	–0.26	.10	.03	[–0.49, –0.02]

	PBWs	–0.81	.09	<.001	[–1.02, –0.60]

TBWs	PBWs	–0.55	.08	<.001	[–0.73, –0.36]

BWESQ – desire/savouring					

NBWs	TBWs	–0.34	.09	.001	[–0.57, –0.12]

	PBWs	–0.62	.08	<.001	[–0.81, –0.41]

TBWs	PBWs	–0.27	.07	.001	[–0.44, –0.09]

BWESQ – engagement					

NBWs	TBWs	–0.32	.09	.002	[–0.54, –0.10]

	PBWs	–0.53	.08	<.001	[–0.73, –0.34]

TBWs	PBWs	–0.21	.07	.01	[–0.38, –0.04]

BWESQ – pleasure preservation					

NBWs	TBWs	–0.13	.13	.57	[–0.43, .17]

	PBWs	–0.40	.11	.002	[–0.67, –0.13]

TBWs	PBWs	–0.27	.10	.02	[–0.51, –0.03]

PANAS – negative affect					

NBWs	TBWs	–0.52	1.20	.90	[–3.34, 2.30]

	PBWs	–3.37	1.07	.005	[–5.90, –0.84]

TBWs	PBWs	–2.85	.94	.008	[–5.08, –0.63]

RTMQ – AERT					

NBWs	TBWs	.07	.68	.99	[–1.54, 1.68]

	PBWs	–1.45	.61	.05	[–2.90, –0.1]

TBWs	PBWs	–1.53	.54	.01	[–2.80, –0.25]

WTMSQ – emotional enhancement					

NBWs	TBWs	–0.11	.10	.55	[–0.36, .14]

	PBWs	–0.25	.09	.02	[–0.47, –0.02]

TBWs	PBWs	–0.14	.08	.22	[–0.33, .06]

**Games-Howell test**.					

BWESQ – dependency					

NBWs	TBWs	–0.03	.07	.89	[–0.19, .13]

	PBWs	–0.38	.07	<.001	[–0.55, –0.21]

TBWs	PBWs	–0.35	.07	<.001	[–0.50, –0.19]

BWESQ – loss of control					

NBWs	TBWs	–0.15	.07	.10	[–0.32, .02]

	PBWs	–0.83	.07	<.001	[–0.99, –0.66]

TBWs	PBWs	–0.68	.07	<.001	[–0.84, –0.52]

BWESQ – positive emotions					

NBWs	TBWs	–0.23	.10	.06	[–0.46, .01]

	PBWs	–0.39	.09	<.001	[–0.61, –0.18]

TBWs	PBWs	–0.17	.07	.04	[–0.33, –0.01]

WTMSQ – coping/escapism					

NBWs	TBWs	–0.19	.09	.10	[–0.41, .03]

	PBWs	–0.54	.08	<.001	[–0.74, –0.34]

TBWs	PBWs	–0.35	.08	<.001	[–0.55, –0.15]


*Note*. AERT: analytic evaluative repetitive thinking; ANOVA: analysis of variance; BWESQ: Binge-Watching Engagement and Symptoms Questionnaire; NBWs: non-binge-watchers; PANAS: Positive and Negative Affect Schedule; PBWs: problematic binge-watchers; RTMQ: Repetitive Thinking Mode Questionnaire; TBWs: trouble-free binge-watchers; WTSMQ: Watching TV Series Motives Questionnaire

#### Binge-watching engagement and symptoms

A main group effect was found for the dimensions binge-watching (F(2, 303) = 50.68, *p* < .001, η^2^ = .25), dependency (F_Welch_(2, 166.98) = 18.15, *p* < .001, η^2^ = .10), desire/savouring (F(2, 303) = 26.80, *p* < .001, η^2^ = .15), engagement (F(2, 303) = 21.29, *p* < .001, η^2^ = .12), loss of control (F_Welch_(2, 164.06) = 83.15, *p* < .001, η^2^ = .34), pleasure preservation (F(2, 303) = 7.53, *p* = .001, η^2^ = .05) and positive emotions (F_Welch_(2, 133.33) = 10.68, *p* < .001, η^2^ = .08). The three groups differed significantly for binge-watching, desire/savouring and engagement, with higher mean scores for PBWs and lower mean scores for NBWs. Regarding dependency, loss of control, pleasure preservation and positive emotions, PBWs scored on average higher than did NBWs and TBWs. Since groups differed significantly solely on binge-watching habits variables, one can conclude that the groups constitute well-matched samples.

#### Rumination

We found a main group effect for AERT (F(2, 303) = 5.23, *p* = .006, η^2^ = .03) but not for CDRT (F(2, 303) = 1.98, *p* = .14, η^2^ = .01) and CERT (F(2, 303) = 3.01, *p* = .051, η^2^ = .02). PBWs reported more AERT than did NBWs and TBWs.

#### Affect

A main group effect was found for negative affect (F(2, 303) = 7.28, *p* = .001, η^2^ = .05), PBWs reporting more negative affect than both NBWs and TBWs. No main group effect was found for positive affect (F(2, 303) = 2.47, *p* = .09, η^2^ = .02).

### Mediation analyses

We conducted mediation analyses because both the total effect (i.e., negative affect à BWESQ or WTSMQ facet) and the indirect effect between negative affect and AERT (β = .33, *p* < .001) were significant.

We found that AERT partially moderated the relationship between negative affect and the coping/escapism dimension of the WTSMQ and fully accounted for the relationship between negative affect and the positive emotions facet of the BWESQ. AERT did not account for any other relationship. These results are reported in Table 4.

**Table 4:** Mediation coefficients and statistical outputs of the total, indirect and direct effects.


DEPENDENT VARIABLE	TOTAL EFFECT (C)				INDIRECT EFFECT (AXB)			DIRECT EFFECT (C’)			

	B	SE	β	p	B	SE	p	B	SE	β	p
		
BWESQ – binge- watching	.02	.01	.28	<.001	.0004	.005	.93	.02	.01	.27	.006

BWESQ – dependency	.03	.01	.40	<.001	.01	.005	.24	.03	.01	.33	<.001

BWESQ – desire/savouring	.02	.01	.25	.002	–.005	.004	.20	.02	.01	.33	.001

BWESQ – engagement	.01	.01	.20	.01	.01	.005	.07	.01	.01	.083	.40

BWESQ – loss of control	.02	.01	.29	<.001	.003	.004	.47	.02	.01	.25	.01

BWESQ – pleasure preservation	.02	.01	.21	.007	.01	.01	.10	.01	.01	.11	.27

BWESQ – positive emotions	.01	.005	.20	.01	.01	.004	.04	.005	.01	.08	.43

WTMSQ –coping/escapism	.05	.01	.53	<.001	.01	.01	.002	.03	.01	.36	<.001


*Note*. BWESQ: Binge-Watching Engagement and Symptoms Questionnaire; Indirect effect: negative affect à AERT à facet of the BWESQ/WTSMQ; Direct effect: negative affect à facet of the BWESQ/WTSMQ; SE: standard error; WTSMQ: Watching TV Series Motives Questionnaire; Total effect: negative affect à facet of the BWESQ/WTSMQ

## Discussion

From a process-based perspective (Kinderman, 2005; Kinderman & Tai, 2007), this study tested the mediating role of ruminative thinking styles on the relationship between negative affect and problematic binge-watching in three different groups of problematic and non-problematic TV series viewers (based on Flayelle et al., 2020b).

We hypothesized, first, that PBWs would 1a) differ from TBWs on binge-watching related motives (with PBWs reporting more negative reinforcement motives) and engagement (with PBWs reporting more symptoms of problematic binge-watching) and 1b) report more negative affect than NBWs and TBWs. Results supported the first hypothesis aspect (i.e., 1a) as PBWs scored higher than TBWs on the coping/escapism motive for binge-watching and all BWESQ dimensions, independently of whether they were associated with more hedonistic (e.g., pleasure preservation) or negative (e.g., loss of control) aspects of binge-watching. Problematic binge-watching thus appears to be characterized by an overgeneralized higher intensity of binge-watching, encompassing the core form of this behaviour (e.g., higher score at the binge-watching facet) and its related positive (e.g., emotional enhancement) or negative (e.g., dependency) affect. In line with the second hypothesis aspect (i.e., 1b), we also found that PBWs reported more negative affect than did NBWs and TBWs. This follows previous empirical evidence of higher self-report of negative affect among problematic binge-watchers (e.g., Flayelle et al., 2019b) and with the conceptualization of this behaviour as a potential means to escape negative emotions (e.g., Panda & Pandey, 2017). Such a view is further supported by PBWs’ higher report of a coping/escapism motive for binge-watching. These results provide information about the risk of overpathologizing everyday behaviours when operationalizing binge-watching as an addictive behaviour, regardless of its underlying processes (Billieux et al., 2015a; Flayelle et al., 2019a; Ort et al., 2021). Indeed, after motives and engagement in binge-watching are taken into consideration, PBWs and TBWs pole apart, whereas TBWs differ from NBWs only on a core characteristic of this behaviour (i.e., binge-watching facet) and its associated positive outcomes (i.e., desire/savouring and engagement dimensions). This falls, however, within the above-mentioned evidence that the binge-watching pattern of viewing TV series is associated with a set of positive outcomes (e.g., narrative transportation; Erickson et al., 2019).

Second, we hypothesized, following the recent triadic conceptualization of rumination (Philippot et al., 2021), that PBWs would principally report an unconstructive ruminative thinking style (i.e., AERT), and that NBWs and TBWs would mainly report constructive ruminative thinking styles (i.e., CERT and CDRT). Although PBWs indeed reported more AERT than did NBWs and TBWs, we found no further group differences regarding CERT and CDRT. This result echoes previous research showing that a ruminative thinking style is present in many mental disorders, including schizophrenia (e.g., overrepresentation of AERT; Maurage et al., 2017), alcohol use disorder (e.g., patients report more AERT than do non-clinical social drinkers; Devynck et al., 2017) and depressive mood (e.g., depressive mood scores are positively associated with AERT in elderly people; Philippot & Agrigoroaei, 2017). Increasing evidence also demonstrates positive associations between a ruminative thinking style and dysregulated behaviours such as problematic smartphone use (Elhai et al., 2020b) or gambling disorder (Ruiz de Lara et al., 2019). Nevertheless, contrary to previous findings linking a constructive repetitive thinking style with a set of positive mental and physical outcomes (for a review, see Watkins, 2008), we did not find any main group effect regarding constructive rumination. Such a lack of group differences does not, however, necessarily go against reports of the prevalence of constructive rumination in pathological populations, because results are inconsistent regarding the hypothesized protective role of constructive rumination in these populations. For example, it has been reported in alcohol use disorder that recently detoxified alcohol-dependent patients did not differ on self-reported CERT—whereas they reported more AERT than did healthy control participants (Grynberg et al., 2016).

Third, we hypothesized that negative affect would be related to problematic binge-watching in PBWs and that AERT would mediate such a relationship. We found that increased negative affect in PBWs was positively associated with the coping/escapism motive and each of the seven facets of the BWESQ, for which PBWs scored higher than NBWs and TBWs. However, the mediating role of AERT was observed for only two specific facets, as AERT 1) partially mediated the relationship between negative affect and the coping/escapism motive and 2) completely mediated the relationship between negative affect and the positive emotions facet (i.e., positive emotions derived from TV series watching) of the BWESQ. Echoing these findings, the mediating role of rumination has also been highlighted in the context of other problematic behaviours, such as problematic involvement in social media (e.g., Dempsey et al., 2019; Mitra & Rangaswamy, 2019) and problematic mobile phone use (e.g., Liu et al., 2017). However, comparison with these previous studies is complicated because some of them considered the problematic/addictive behaviour as the antecedent (i.e., risk factor). Moreover, variables of interest almost always differed among studies (i.e., different behaviours, risk factors and psychological processes investigated).

The mediation analyses in the current study offer some noteworthy findings. All associations—whether significant or not—were positive, with the exception of the small indirect effect between negative affect, AERT and desire/savouring. This suggests that problematic binge-watching is associated with higher engagement and symptom intensity, regardless of their valence. Moreover, the current results imply that, when experiencing negative affect, PBWs’ viewing behaviour is underpinned by coping/escapism-reasoning, partially through an unconstructive rumination effect. It also seems that a higher self-reported experience of negative affect is related to a higher report of positive emotions in PBWs—solely through an unconstructive rumination effect. Together, these results therefore indicate that binge-watching might serve as a maladaptive coping behaviour in which problematic binge-watchers, when facing distressful emotional states, binge-watch to aim for mood enhancement, with an unconstructive ruminative thinking style acting as a relevant mediating psychological process in this context.

In uncovering the underlying role of unconstructive rumination in the onset of problematic binge-watching, the present results have clinical implications. For example, rumination-centred therapies might be of particular interest in the behavioural field (e.g., de Lisle et al., 2012). Although reports of therapeutic interventions that target an unconstructive ruminative thinking style to reduce the production of problematic behaviours are few, studies investigating mindfulness-related processes suggest that such an approach is effective (e.g., a higher level of mindfulness associated with a lower level of rumination and, subsequently, a lower level of smartphone addiction severity in Cheng et al., 2020).

Several limitations should be acknowledged. First, all measures were self-reported, potentially generating response biases (e.g., discrepancy between perceived and actual device use; Araujo et al., 2017). Second, the high proportion of female participants (74.60% to 84.70%) might have affected the current results, as women are known to be more prone than men are to negative affect and to engage more than men do in ruminative coping (e.g., Nolen-Hoeksema et al., 1999). Third, although our group criteria were based on previous experimental work (Flayelle et al., 2020b), there remains a need to develop empirically validated criteria for binge-watching operationalization (see Flayelle et al., 2020a and Starosta & Izydorczyk, 2020). Fourth, the current triadic approach of rumination is based on preliminary evidence (Philippot et al., 2021), which, although based on the previous dualistic approach of rumination (Watkins, 2008), needs further research to ascertain its validity. Finally, future longitudinal studies are required to overcome the limits linked to cross-sectional designs, notably to confirm the soundness of our process-based approach of problematic binge-watching.

By capitalizing on a process-based approach to test the mediating role of unconstructive rumination on the relation between negative affect and problematic binge-watching, our study fosters the elucidation of the psychological processes at play in problematic binge-watching, therefore paving the way to a better understanding of its aetiology and hence therapeutic avenues. Investigating the processes underlying binge-watching might also prevent overpathologization of such a prevalent leisure activity. Furthermore, our results highlight the necessity of differentiating trouble-free from problematic binge-watching as, apart from the binge-watching behaviours themselves, trouble-free binge-watchers seem to share more characteristics with non-binge-watchers than with problematic binge-watchers.

This original research paper was submitted in the context of the special issue in honour of Martial Van der Linden as he was a pioneer in promoting process-based approach to psychopathology and clinical psychology. Inspired by the ground-breaking work of Peter Kinderman (Kinderman, 2005; Kinderman et al., 2013), Martial has been extensively involved in teaching the process-based approach and making this framework available to French-speaking undergraduates and clinical psychologists. With passion and commitment, Martial also transmitted the core principles of this approach to his colleagues and PhD students, making them using it in their clinical practice and research (see e.g. Billieux et al., 2015b). Martial definitely plays a major role in the fact that process-based approach to case conceptualization and psychological intervention is now taught at the master and post-grad levels in the University of Lausanne (UNIL), the University of Geneva (UNIGE), the University of Liege (ULG), or the Catholic University of Louvain (UCLouvain).

## Footnotes

These variables were assessed by means of the two following questions: “Does TV series watching have already negatively impacted your everyday life (e.g., sleep deprivation, postponing of daily tasks, displacement of other activities, close relatives’ reproaches, etc)?”, “Do you consider your TV series consumption as problematic?” (Flayelle et al., 2020b).
